# *Corynebacterium diphtheriae* Infections, South Africa, 2015–2023

**DOI:** 10.3201/eid3103.241211

**Published:** 2025-03

**Authors:** Mignon du Plessis, Rito Mikhari, Linda de Gouveia, Noluthando Duma, Tamsin Lovelock, Charlene Lawrence, Prasha Mahabeer, Yesholata Mahabeer, Nevashan Govender, Susan Nzenze, Jonathan Featherston, Mishalan Moodley, Jocelyn Moyes, Sibongile Walaza, Cheryl Cohen, Anne von Gottberg

**Affiliations:** National Health Laboratory Service, Johannesburg, South Africa (M. du Plessis, R. Mikhari, L. de Gouveia, N. Duma, N. Govender, S. Nzenze, J. Featherston, M. Moodley, J. Moyes, S. Walaza, C. Cohen, A. von Gottberg); University of the Witwatersrand, Johannesburg (M. du Plessis, R. Mikhari, S. Walaza, C. Cohen, A. von Gottberg); Stellenbosch University and Tygerberg Hospital, Cape Town, South Africa (T. Lovelock); Western Cape Department of Health, Cape Town (C. Lawrence); National Health Laboratory Service, Durban, South Africa (P. Mahabeer, Y. Mahabeer); University of Kwazulu-Natal, Durban (P. Mahabeer, Y. Mahabeer); University of Cape Town, Cape Town (A. von Gottberg)

**Keywords:** *Corynebacterium diphtheriae*, diphtheria, cutaneous diphtheria, infective endocarditis, molecular epidemiology, DTP vaccine, diphtheria toxin, *tox* gene, genome, antimicrobial resistance, bacteria, South Africa, *Suggested citation for this article*: du Plessis M, Mikhari R, de Gouveia L, Duma N, Lovelock T, Lawrence C, et al. *Corynebacterium diphtheriae* infections, South Africa, 2015–2023. Emerg Infect Dis. 2025 Mar [*date cited*]. https://doi.org/10.3201/eid3103.241211

## Abstract

We reviewed *Corynebacterium* spp. infection cases reported in South Africa during 2015–2023. We analyzed 84 isolates from 83 patients with *C. diphtheriae*, as well as 1 *C. belfantii* and 3 *C. ulcerans* isolates. Among *C. diphtheriae* cases, we observed respiratory diphtheria (26/83 patients [31%]), endocarditis (14/83 [17%]), cutaneous diphtheria (22/83 [27%]), nonspecific respiratory illnesses (5/83 [6%]), and asymptomatic carriage (16/83 [19%]). The median patient age was 19 (range 0–88) years. Diphtheria-tetanus-pertussis vaccination was incomplete for 26% (5/19) or unknown for 68% (13/19) of children 0–9 years of age. *C. diphtheriae* was intermediately resistant to penicillin (82/84 [98%] isolates; MIC_90_ 0.5 μg/mL) but susceptible to erythromycin (83/84 [99%] isolates; MIC_90_ 0.25 μg/mL). Eighteen unique sequence types were identified, corroborating *C. diphtheriae* heterogeneity. Toxin-producing strains were detected among cutaneous and respiratory diphtheria cases, indicating all forms of disease require monitoring and prompt public health action to curb transmission.

Diphtheria is a potentially fatal disease caused by toxigenic strains of *Corynebacterium diphtheriae*, *C. ulcerans*, or *C. pseudotuberculosis.* Diphtheria-tetanus-pertussis (DTP) vaccination has led to declines in the global incidence of diphtheria. However, since the early 1990s, a global resurgence in *C. diphtheriae* infections has occurred. Since 2023, an increase in diphtheria cases has been recorded in 4 countries (Guinea, Mauritania, Niger, Nigeria) in Africa, all of which have been experiencing ongoing, active outbreaks (*1*).

Resurgence of diphtheria has been caused by several factors, including disruptions in vaccination programs in countries with low socioeconomic status or political instability (*2*,*3*), increased awareness and reporting of nontoxigenic infections (*4*,*5*), and changing epidemiology in some settings (*6*). Adolescents and adults whose vaccine-induced or naturally induced protection wanes in the absence of sustained transmission of toxigenic strains or adequate booster immunization are particularly vulnerable during diphtheria outbreaks (*7*). Vaccine coverage of 80%–85% has been previously recommended to maintain herd immunity at the population level (*8*); however, more recent data recommend a coverage threshold of >90% (*9*).

Diphtheria toxin is the primary virulence factor in toxigenic *Corynebacterium* spp., inhibiting protein synthesis in target host cells (*10*). The phage-encoded toxin gene, *tox*, integrates into the bacterial genome by site-specific recombination. Nontoxigenic *C. diphtheriae* can produce toxin if they are lysogenized with a toxin gene–carrying corynephage. Some nontoxigenic *C. diphtheriae* isolates harbor the *tox* gene but are not able to express toxin because of a frameshift mutation or insertion sequence in this gene (referred to as nontoxigenic, toxin gene–bearing [NTTB] *C. diphtheriae*) (*11*). Although rare, NTTB *C. diphtheriae* has been reported as an emerging pathogen in some countries (*11*,*12*).

Classical respiratory diphtheria caused by toxigenic *Corynebacterium* strains is characterized by sore throat, low-grade fever, a swollen neck, and the presence of a gray/white pseudomembrane covering the tonsils, pharynx, or larynx that can cause airway obstruction and suffocation. Reports of invasive infections caused by nontoxigenic *C. diphtheriae* have notably increased and can manifest as bacteremia, endocarditis, and other more unusual clinical syndromes (*13*,*14*). Cutaneous diphtheria, also caused by *C. diphtheriae* (toxigenic or nontoxigenic) in skin lesions or nonhealing ulcers, is often less severe but might serve as a potential reservoir for transmission of toxigenic and nontoxigenic *C. diphtheriae* (*15*).

Treatment for toxigenic diphtheria involves administering of diphtheria antitoxin (DAT) to neutralize circulating toxin and antimicrobial drugs (β-lactams or macrolides) to eradicate the bacterium in patients and close contacts. However, a global shortage of DAT and bacterial resistance to first-line antimicrobial drugs have been reported, potentially complicating clinical management of *C. diphtheriae* infections (*16*–*18*). Genomic data can clarify the distribution of resistance determinants and their association with phenotype or lineage. We evaluated characteristics of isolates from reported *C. diphtheriae* infections in South Africa during 2015–2023 by using epidemiologic and molecular methods.

## Methods

### Ethics Approval

Investigations related to notifiable medical conditions, including access to medical records, are allowable in South Africa under the terms of the National Health Act 2003 (Act No. 61 of 2003): Regulations Relating to the Surveillance and Control of Notifiable Medical Conditions. Furthermore, the South Africa National Institute for Communicable Diseases of the National Health Laboratory Service is subject to oversight by the Human Research Ethics Committee of the University of the Witwatersrand, Johannesburg, regarding the application of good clinical and laboratory practice while serving the interests of public health in the collection, analysis, and interpretation of communicable diseases data (ethics certification no. M160667).

### Disease Classification/Category

Diphtheria is a category 1 legally notifiable medical condition in South Africa. Diagnostic laboratories send clinical specimens and isolates of *C. diphtheriae*, *C.* *ulcerans*, and *C. pseudotuberculosis* from patients with suspected respiratory or cutaneous diphtheria, or any other clinical manifestation, to the national reference laboratory for confirmation and toxin production analysis.

We classified infections as respiratory diphtheria (detection of toxigenic *C. diphtheriae/ulcerans/pseudotuberculosis* in nose or throat samples of patients with respiratory illness), cutaneous diphtheria (detection of toxigenic or nontoxigenic *C. diphtheriae/ulcerans/pseudotuberculosis* in a nonhealing ulcer or wound), or endocarditis (detection of *C. diphtheriae* in blood and clinical signs compatible with endocarditis). We classified patients with nonspecific respiratory disease as those with nontoxigenic *C. diphtheriae* infections incidentally isolated during routine microbiology laboratory workup. We classified persons as asymptomatic if they were carriers of *C. diphtheriae* (in the nose or throat) and in close contact with symptomatic patients who had laboratory-confirmed *C. diphtheriae* infections.

### Laboratory Methods

We confirmed species identification of isolates by using matrix-assisted laser desorption/ionization time-of-flight mass spectrometry (*19*); we used a Microflex LT/SH analyzer with FlexControl version 3.4.135 and FlexAnalysis version 3.4.76.00 software (Bruker Daltonics, https://www.bruker.com). In addition, we performed PCR to identify the *rpoB* gene specific for *C. diphtheriae*, the *rpoB* gene specific for *C. ulcerans*/*pseudotuberculosis*, and the *tox* gene for all 3 species (*20*). We used a modified Elek test to measure toxin production (*21*). We performed antimicrobial susceptibility testing by using the broth microdilution method according to Clinical and Laboratory Standards Institute (CLSI) guidelines (*22*). We used Sensititer STP6F MIC panels (Thermo Fisher Scientific, https://www.thermofisher.com) to test susceptibility to 20 antimicrobial drugs (Appendix 1 Table). We used the API Coryne kit (bioMérieux, https://www.biomerieux.com) to measure nitrate reduction. When they were available, we extracted basic patient demographic and clinical data from medical records, including year of symptom onset, patient sex, region (province), specimen type, clinical diagnosis, DTP vaccination history, hospitalization, and outcome.

### Genome Sequencing and Characterization

We extracted and sequenced DNA from *C. diphtheriae* as previously described (*23*) by using an Illumina NextSeq 1000/2000 instrument (Illumina, https://www.illumina.com); coverage depth was >100×. We trimmed raw reads by using Trim Galore version 0.6.2 (Babraham Bioinformatics, https://www.bioinformatics.babraham.ac.uk) and de novo assembled the reads by using SPAdes version 3.12.0 (*24*). We performed assembly quality checks by using BUSCO version 5.8; assembly completeness of >90% was the cutoff for inclusion (*25*). We deposited raw sequences in the National Center for Biotechnology Information BioSample database (https://www.ncbi.nlm.nih.gov/biosample; accession nos. SAMN45099837–922) (Appendix 1 Table). We submitted assembled genomes to the Insitut Pasteur Bacterial Isolate Genome Sequence *C. diphtheriae* database (https://bigsdb.pasteur.fr/diphtheria) for curation and sequence type (ST) assignments. We used core genome multilocus sequence typing (cgMLST) of 1,305 loci for sublineage (SL) classification within the database by using a 500-allelic mismatch threshold (*26*,*27*).

We analyzed genomic features, such as antimicrobial resistance genes (*pbp2m* for penicillin and *ermX* for erythromycin resistance), biovar (the presence of the *spuA* gene [DIP0357 locus] indicated biovar gravis; absence of *spuA* indicated biovar mitis), and known virulence genes, by using the diphtOscan framework with assembled genomes as inputs (*17*,*27*). To verify the presence or absence of antimicrobial resistance genes, we scanned raw reads by using DeepARG version 1.0.4 after converting reads from fastq format to fasta with SeqKit (*28*,*29*).

### Phylogeny

Using JolyTree version 2.1, we generated an alignment-free, distance-based tree for phylogenetic inference of 84 assembled genomes (*30*) and 2 additional genomes from clinical isolates collected in South Africa during the 1980s (for which no clinical or demographic data were available). We used the tree alongside a cgMLST-based MAFFT alignment generated by using Genome Comparator to serve as input for ClonalFrameML version 1.2 (*31*); we visualized and annotated the tree by using iTOL (https://itol.embl.de). To enhance resolution among outbreak clusters, we determined single-nucleotide polymorphisms (SNPs) and SNP distances by mapping assembled reads of each genome to a *C. diphtheriae* reference strain (GenBank accession no. NCTC13129) by using the Split Kmer analysis tool (S.R. Harris, unpub. data, https://doi.org/10.1101/453142).

## Results

During the study period, 83 *C. diphtheriae*, 1 *C. belfantii*, and 3 *C. ulcerans* infection cases were reported nationally. No cases of *C. pseudotuberculosis* were reported.

### *C. diphtheriae* Infections

**Figure 1:**
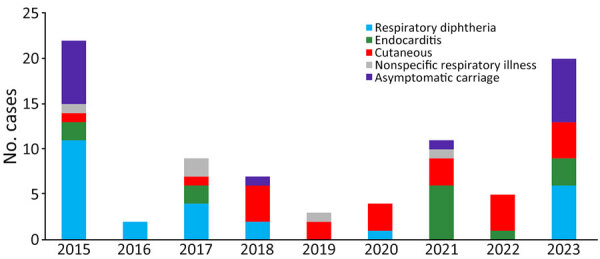
*Corynebacterium diphtheriae* infections according to year and clinical illness category, South Africa, 2015–2023. Total number of cases was 83.

The clinical categories for 83 *C. diphtheriae* culture-positive cases were as follows: toxigenic respiratory diphtheria (26/83 [31%] patients), cutaneous diphtheria (22/83 [27%]), nontoxigenic infective endocarditis (14/83 [17%]), asymptomatic (16/83 [19%]), and nonspecific respiratory illness (5/83 [6%]) (Table 1; Figure 1). Of the 83 patients, 50 (61%) were male and 32 (39%) female; sex was not recorded for 1 person. Median age was 19 years (range 6 months–88 years). DTP vaccination status was incomplete for 26% (5/19) or unknown for 68% (13/19) of children <10 years of age (only 1 child was fully vaccinated). One patient’s throat was colonized with 2 different strains (*23*), resulting in a total of 84 *C. diphtheriae* cultures. PCR and culture results were 100% concordant for all samples. The Elek tests correlated with PCR *tox* gene results for all cultures; no NTTB isolates were identified.

**Table 1:** Characteristics of *Corynebacterium diphtheriae* infection cases, South Africa, 2015–2023*

Characteristics	Respiratory diphtheria	Endocarditis	Cutaneous diphtheria	Nonspecific respiratory illness	Asymptomatic
No. patients/group	26	14	22	5	16
Toxin positive	26 (100)	0	2 (9)	0	14 (88)
Year of bacteria isolation					
2015	11 (42)	2 (14)	1 (5)	1 (20)	7 (44)
2016	2 (8)	0	0	0	0
2017	4 (15)	2 (14)	1 (5)	2 (40)	0
2018	2 (8)	0	4 (18)	0	1 (6)
2019	0	0	2 (9)	1 (20)	0
2020	1 (4)	0	3 (14)	0	0
2021	0	6 (43)	3 (14)	1 (20)	1 (6)
2022	0	1 (7)	4 (18)	0	0
2023	6 (23)	3 (21)	4 (18)	0	7 (44)
Province					
Gauteng	0	0	3 (14)	1 (20)	0
Western Cape	9 (35)	14 (100)	2 (9)	2 (40)	8 (50)
Eastern Cape	0	0	12 (55)	1 (20)	0
KwaZulu-Natal	17 (65)	0	4 (18)	1 (20)	8 (50)
North West	0	0	1 (5)	0	0
Age category, y					
0–4	3 (12)	0	0	2 (40)	1 (6)
5–9	4 (15)	4 (29)	0	0	4 (25)
10–19	10 (38	5 (36)	2 (9)	1 (20)	6 (38)
20–45	9 (35)	5 (36)	12 (55)	2 (40)	5 (31)
>45	0	0	8 (36)	0	0
Patient sex					
M	16 (62)	9 (64)	14 (64)	2 (50)†	9 (56)
F	10 (38)	5 (36)	8 (36)	2 (50)	7 (44)
Outcome					
Died	8 (31)	6 (43)	0	0	0
Survived	15 (58)	4 (29)	9 (41)	1 (20)	16 (100)
Unknown	3 (12)	4 (29)	13 (59)	4 (80)	0
Hospitalization					
Inpatient	23 (88)	14 (100)	11 (50)	2 (40)	0
Outpatient	2 (8)	0	10 (45)	2 (40)	16 (100)
Unknown	1 (4)	0	1 (5)	1 (20)	0
Vaccine history					
Fully vaccinated for age	1 (4)	1	0	1 (20)	0
Incomplete/unvaccinated	7 (27)	0	0	0	0
Unknown/not recorded	18 (69)	13 (93)	22 (100)	4 (80)	16 (100)

*Values are no. (%). Total number of cases was 83. †Sex unknown for 1 person.

### Other *Corynebacterium* spp. Infections

Toxin-producing *C. ulcerans* was detected in 1 patient >65 years of age who had suspected diphtheria in 2016. In 2017, *C. ulcerans* was reported in an elderly patient with a pituitary adenoma; however, that isolate was not available for further characterization. In 2020, nontoxigenic *C. ulcerans* was isolated from a uterine tissue sample from a 37-year-old patient with a history of miscarriage. No information regarding animal exposure, outcome, or DAT administration was available for *C. ulcerans* cases.

*C. belfantii* (nontoxigenic) was isolated in 2023 from a sputum sample from an elderly patient with nonspecific respiratory illness. We identified the isolate as *C. diphtheriae* by using mass spectrometry and PCR. We classified the isolate as *C. belfantii* according to the absence of nitrate reductase genes and corresponding inability to reduce nitrates, characteristic of *C. belfantii* (*32*). Because *C. belfantii* has been reclassified from a biovar to a separate *Corynebacterium* species (*33*), we excluded this species from the *C. diphtheriae* dataset.

### Respiratory Diphtheria

**Figure 2:**
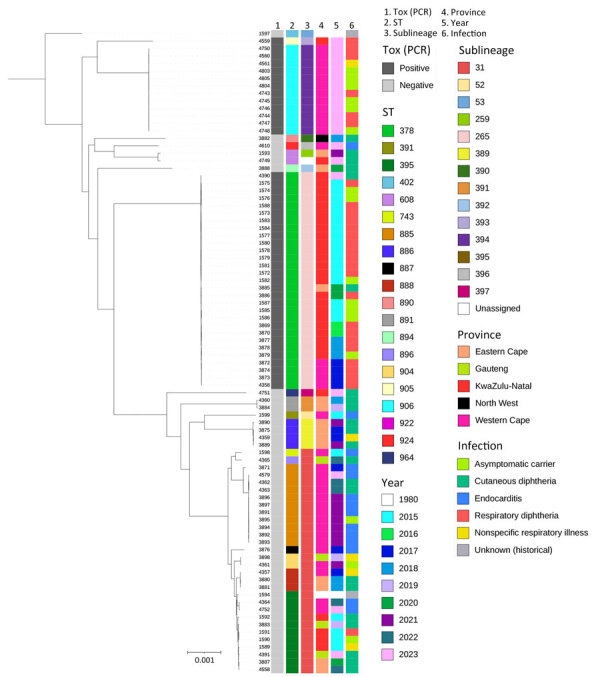
Phylogenetic analysis of *Corynebacterium diphtheriae* isolates, South Africa, 2015–2023. Total number of isolates was 84 from 83 patients. Isolate identification numbers are listed on the right side of the colored bars. Colored columns indicate presence/absence of the *tox* gene, sequence type, sublineage, location of isolate, year isolate was collected, and clinical infection type. Neighbor-joining tree was generated by using the core genome multilocus sequence typing scheme in the Insitut Pasteur Bacterial Isolate Genome Sequence *C. diphtheriae* database (https://bigsdb.pasteur.fr/diphtheria). Tree was visualized by using iTOL (https://itol.embl.de) and rooted by using a *tox* gene–negative *C. diphtheriae* genome (no. 1597 at top) isolated from South Africa circa 1980 (clinical isolate with no available clinical or demographic data). Scale bar indicates nucleotide substitutions per site. ST, sequence type.

Respiratory diphtheria was diagnosed in 26 patients. The case-fatality ratio among *C. diphtheriae* cases with known outcomes was 35% (8/23) (Table 1). Eleven cases, all toxigenic ST378, were from a community outbreak in KwaZulu-Natal during 2015 (*23*,*34*). A second cluster of 3 diphtheria cases occurred in a correctional services facility in the Western Cape in 2023, caused by toxigenic ST906. The median patient ages were 10 (range 4–41) years in KwaZulu-Natal and 19 (range 18–20) years in Western Cape. The remaining 12 diphtheria cases were sporadic and occurred in the same 2 provinces; bacteria strains were identified as ST378 (n = 9), ST905 (n = 1), and ST906 (n = 2) (Table 2; Figure 2).

**Table 2:** Clinical characteristics of *Corynebacterium diphtheriae* isolates, South Africa, 2015–2023*

Clinical category	No. isolates/total (%)	Sequence type/sublineage†
Total no. isolates	84	NA
Respiratory diphtheria	27/84 (32)	NA
Toxin positive	26/27 (96)	ST378/SL265, n = 20; ST905/SL393, n = 1; ST906/SL394, n = 5
Toxin negative‡	1/27 (4)	ST395/SL31, n = 1
Endocarditis	14/84 (17)	NA
Toxin positive	0	NA
Toxin negative	14/14 (100)	ST391/SL52, n = 1; ST395/SL31, n = 2; ST743/SL31, n = 1; ST885/SL31, n = 8; ST887/SL31, n = 1; ST924/SL396, n = 1
Cutaneous diphtheria	22/84 (26)	NA
Toxin positive	2/22 (9)	ST378/SL265, n = 2
Toxin negative	20/22 (91)	ST395/SL31, n = 5; ST608/SL259, n = 2§; ST885/SL31, n = 2; ST886/SL389, n = 3; ST888/SL31, n = 2; ST890/SL390, n = 1; ST891/SL391, n = 2; ST894/SL392, n = 1; ST896/SL31, n = 1; ST964/SL397, n = 1
Nonspecific respiratory illness	5/84 (6)	NA
Toxin positive	0	NA
Toxin negative	5/5 (100)	ST395/SL31, n = 1; ST886/SL389, n = 1; ST888/SL31, n = 1; ST904/SL31, n = 2
Asymptomatic carrier¶	16/84 (19)	NA
Toxin positive	14/16 (88)	ST378/SL265, n = 7; ST906/SL394, n = 7
Toxin negative	2/16 (13)	ST395/SL31, n = 1; ST885/SL31, n = 1

*Total number of isolates was 84 from 83 patients. NA, not applicable; SL, sublineage; ST, sequence type. †Sublineage identified by using core genome multilocus sequence typing (*26*). ‡One patient with respiratory diphtheria harbored toxigenic ST378 and nontoxigenic ST395 in their throat. §Sublineage not assigned for 1 isolate. ¶Contacts of symptomatic patients (did not develop respiratory symptoms).

### Infective Endocarditis

Endocarditis cases (n = 14) were caused by nontoxigenic *C. diphtheriae*; the case-fatality ratio was 60% (6/10) among patients with known outcomes (Table 1). The median patient age was 14 (range 5–38) years, and all cases were reported from the Western Cape. Five of those cases were geographically and temporally linked, and detailed clinical aspects have been previously described (*35*); 1 patient from the cluster reported substance abuse (not intravenous), 1 had undergone a mitral valve replacement, and the remaining 3 did not have a known underlying illness or report a history of substance/alcohol abuse. Among the remaining 9 endocarditis cases, 5 patients had underlying illness or were substance abusers; underlying illnesses were not captured for 4 of those patients. Although 6 STs were identified, most (57% [8/14]) cases were caused by *C. diphtheriae* ST885 (Table 2; Figure 2).

### Cutaneous Diphtheria

Cutaneous diphtheria accounted for 27% (22/83) of *C. diphtheriae* infections, reported from 5 of 9 provinces (Table 1). The median patient age was 38 (range 15–88) years. Two cases, reported in 2020 (Eastern Cape) and 2023 (KwaZulu-Natal), were caused by toxigenic ST378. The other 20 cases were a mixture of 10 nontoxigenic (mostly unrelated) STs (Table 2; Figure 2).

### Nonspecific Respiratory Illness and Asymptomatic Carriers

Incidental isolation of nontoxigenic *C. diphtheriae* was reported in 5/83 (6%) patients during routine diagnostic testing, representing 4 different sequence types; *C. diphtheriae* was isolated from 16/83 (19%) asymptomatic contacts of symptomatic patients who had laboratory-confirmed *C. diphtheriae* (Tables 1, 2)*.* During the outbreak investigations, *C. diphtheriae* was isolated from 8/145 (6%) close contacts in KwaZulu-Natal during 2015 and 6/151 (4%) close contacts in Western Cape during 2023. During the KwaZulu-Natal outbreak, 6/8 (75%) asymptomatic contacts carried the toxigenic outbreak strain (ST378) in their throats; during the Western Cape outbreak, all asymptomatic contacts carried the same toxigenic strain (ST906) as the symptomatic patients. Asymptomatic contacts did not develop respiratory symptoms.

### Antimicrobial Susceptibility Profiles 

Almost all *C. diphtheriae* isolates were intermediately resistant to penicillin (82/84 [98%]), amoxicillin (83/84 [99%]), and cefotaxime (83/84 [99%]) (Appendix 1 Table). For penicillin, MIC_50_ was 0.25 μg/mL and MIC_90_ was 0.5 μg/mL. For both amoxicillin and cefotaxime, MIC_50_ and MIC_90_ were 2 μg/mL. Eleven (13%) isolates were intermediately resistant to tetracycline (MIC 8 μg/mL) and belonged to lineage ST885/SL31. All isolates were susceptible to linezolid, meropenem, and vancomycin. The 2 isolates from 1980 were susceptible to penicillin, amoxicillin, and cefotaxime (penicillin, MIC 0.03 μg/mL; amoxicillin and cefotaxime, MIC 0.12 μg/mL). Four nontoxigenic isolates belonging to different lineages were nonsusceptible to >3 drug classes. *C. diphtheriae* from 1 fatal case of infective endocarditis was nonsusceptible to 5 antimicrobial drugs, including penicillin (MIC 0.25 μg/mL) and erythromycin (MIC 2 μg/mL), and was the only isolate that was nonsusceptible to erythromycin and also harbored the *pbp2m* gene.

### *C. diphtheriae* Population Structure and Phylogeny

We identified 18 novel STs among 84 genomes from 83 patients (Table 2; Figure 2). The most prevalent STs were toxigenic ST378 (29/84 [35%] isolates) and ST906 (12/84 [14%]) and nontoxigenic ST885 (11/84 [13%]) and ST395 (10/84 [12%]). Toxigenic and nontoxigenic isolates had mutually exclusive STs with no overlap. We identified 12 SLs among 83 isolates (an SL was not assigned for 1 isolate because of poor sequence quality) by using cgMLST (Table 2). SL265 (29/83 [35%]) was exclusively found in ST378 isolates, and SL394 (12/83 [14%]) was only found in ST906 isolates. Pairwise SNP distances were <100 SNPs for both ST378 and ST906 isolates. We observed the same pairwise SNP distance for ST885 isolates except for 1 isolate (from 2017), which differed by 1,632–1,646 SNPs from other ST885 isolates (Appendix 2 Table 1).

### *spuA* and Virulence-Associated Genes

PCR and the diphtOscan pipeline confirmed the presence of the *tox* gene in 42/84 (50%) isolates (Appendix 1). We assessed the potential effect of amino acid mutations on toxin structure as previously described (*36*) and identified 3 toxin variants: *tox* gene variant 6 (toxin group 8) associated with ST378 (n = 29), *tox* variant 16 (toxin group 7) associated with ST905 (n = 1), and *tox* variant 29 (not assigned to a toxin group) associated with ST906 (n = 12). *tox* variants 6 and 16 shared a low impact mutation (T262A), and *tox* variant 16 had an additional moderate impact mutation (V233A). Using the *spuA* gene as a proxy for biovar gravis, 20/42 (48%) nontoxigenic isolates harbored *spuA* and represented a mixture of 8 STs. All toxigenic isolates were classified as biovar mitis according to the absence of *spuA*. The *spa*-like pili (adhesin) genes *spaA*, *spaH*, and *spaD* and *chtAB* (iron uptake) were absent from all toxin-positive isolates but were present in most toxin-negative isolates (*spaA*, 37/42 [88%]; *spaH*, 20/42 [48%]; *spaD*, 33/42 [79%]; and *chtAB*, 37/42 [88%]) (Appendix 2 Table 2).

Virulence gene profiles were mostly conserved among isolates representing the predominant, outbreak-associated ST378 and ST 906 (respiratory diphtheria) and ST885 (endocarditis) lineages. The *spaA*, *spaH*, *spaD*, and *chtAB* genes were absent in ST378 and ST906, whereas all (with the exception of *spaH*) were present in ST885 (Appendix 2 Table 3). *Irp2ABCDEFGHI* (siderophore biosynthesis) and iron uptake system genes *irp2JKLMN* and *htaA-hmuTUV-htaBC* were present in all ST378 and ST906 genomes but absent in ST885.

## Discussion

We provide insight into the types, pathogenicity, and characteristics of *C. diphtheriae* infections after their reemergence in South Africa in 2015. Intermediate resistance to penicillin for almost all isolates indicates real-time monitoring of treatment outcomes is critical to identify emerging clinically significant resistance. Infections were caused by diverse and novel genotypes, confirming the genetic heterogeneity and phylogeographic clustering of *C. diphtheriae* described in other countries (*17*,*36*); however, outbreak-associated lineages were highly conserved even among sporadic cases. Patients with cutaneous diphtheria and nonspecific respiratory illness and asymptomatic carriers promote ongoing transmission, providing a reservoir of strains for genetic exchange. The reemergence of diphtheria has increased awareness among clinicians and diagnostic laboratories in South Africa and highlights the importance of surveillance and active case management for all *C. diphtheriae* cases irrespective of clinical symptoms.

Diphtheria-related deaths in our study were higher (6%–24%) than those reported in other settings (*37*,*38*), likely caused by several factors, such as incomplete vaccination and lack of booster doses, delays in seeking healthcare, lack of accurate symptom onset dates, and limited availability and timely administration of DAT. Our findings highlight the lack of systematic data collection (often unknown or not captured in detail). Data collection methods need improvement to properly assess risk factors associated with diphtheria-related deaths in our setting.

Diphtheria outbreaks are usually associated with inadequate vaccination coverage (*39*). During the KwaZulu-Natal community outbreak in 2015, coverage for the primary series of diphtheria vaccinations in the province was high (96%); however, coverage was substantially lower for the 18-month (83%), 6-year (56%), and 12-year (20%) booster doses (*23*). Vaccination coverage during the second diphtheria cluster in the Western Cape in 2021–2023 was >80% for the primary series, declining to <80% for the 18-month dose; tetanus-diphtheria boosters at 6 and 12 years were inadequate at <50% (C. Lawrence, unpub. data). Vaccine coverage in KwaZulu-Natal and the Western Cape was comparable to that in other provinces (*40*), and increased clinical awareness in those 2 regions might have contributed to the higher number of detected cases. The World Health Organization and United Nations Children’s Fund (https://immunizationdata.who.int) have estimated that DTP3 vaccination coverage in South Africa has been consistently >80% since 2014; however, inaccuracies in data reporting and data quality exist in South Africa, and coverage might be lower. Similar to the case for other countries, disruption in immunization services and changes in healthcare-seeking behavior occurred in South Africa during the COVID-19 pandemic. The number of reported cases of *C. diphtheriae* is too low to directly measure the effects of the COVID-19 pandemic. However, transmission of other respiratory pathogens was interrupted because of social distancing and nonpharmaceutical interventions (*41*), which likely holds true for *C. diphtheriae* transmission.

A cluster of geographically linked cases of *C. diphtheriae* endocarditis among young adults in 2021 with a high death rate indicates that nontoxigenic *C. diphtheriae* infections should not be overlooked (*35*). Although infective endocarditis cases are mostly sporadic, outbreaks caused by single clones have been reported and, similar to our cases, risk factors included drug use, homelessness, and underlying illnesses (*42*,*43*).

In South Africa, toxin production confirmation is usually performed at the national reference laboratory, making it possible to monitor all forms of disease and detect other *Corynebacterium* spp. Cutaneous *C. diphtheriae* and *C. ulcerans* cases have been increasingly reported in Europe, partly because of changes in laboratory testing methods and guidelines (*6*). *C. ulcerans* is predominantly zoonotic but can also cause diphtheria-like illness and be toxigenic, requiring treatment and public health actions similar to those used for *C. diphtheriae* infections.

NTTB strains have not been reported in South Africa, and we did not identify clones that had both toxigenic and nontoxigenic properties. Poland and Germany have both reported nontoxigenic ST8 strains isolated from blood, cutaneous, and respiratory tract specimens (*4*,*44*). Toxigenic *C. diphtheriae* ST8 was responsible for the extensive respiratory diphtheria outbreak in the former Soviet Union in the 1990s (*4*,*44*). ST8 has transformed to a less virulent, nontoxigenic variant, which presumably sustains its spread among highly vaccinated populations in Europe. Molecular typing data from Africa are limited, but nontoxigenic and toxigenic isolates with the same genotype (ST377) were recently isolated from 2 immigrants from West Africa who had cutaneous diphtheria (*18*). Those findings stress the importance of monitoring all manifestations of *C. diphtheriae* disease.

In South Africa, diphtheria case management and prophylaxis for close contacts of diphtheria patients involves administering either penicillin or macrolides. Emerging penicillin resistance in different countries prompted the World Health Organization to update its guidelines in 2024 to recommend the use of macrolides in preference to β-lactams (https://www.who.int/teams/health-care-readiness/clinical-management-of-diphtheria). Until recently, MIC breakpoints for antimicrobial resistance have been undefined; however, CLSI updated its guidelines in 2015 to include interpretative criteria to define nonsusceptibility. Interpretation is complicated by different breakpoints to determine penicillin nonsusceptibility (MIC >4 µg/mL in CLSI guidelines and >1 µg/mL in EUCAST guidelines; https://www.eucast.org/clinical_breakpoints). Two genomic studies using geographically representative datasets demonstrated that the *pbp2m* gene correlates with a penicillin-resistant phenotype (*17*,*27*); however, other studies showed intermediate-resistant isolates did not necessarily harbor *pbp2m* (*18*,*45*,*46*). The contribution of other *pbp* genes to β-lactam resistance and increased MICs has not been conclusively established (*46*). Furthermore, the clinical significance of intermediate resistance to penicillin is not fully understood, and it remains critical to monitor treatment failures (for symptomatic cases) and failure to eradicate carriage in close contacts of diphtheria patients.

*C. diphtheriae* is subdivided into biochemically distinct biovars that could be associated with increased severity (*47*). Differentiation can be technically challenging and earlier genomic studies could not confidently differentiate biovars (*48*). Studies have shown concordance between the *spuA* gene and biovar gravis (*17*,*49*). Gravis isolates are largely nontoxigenic (mitis isolates are mostly toxigenic) (*18*), which was consistent with our findings. We did not find a clear distinction among mitis and gravis virulence gene profiles among nontoxigenic isolates in our dataset. We observed an absence of *spa*-type pili genes in toxigenic isolates, which were present in the nontoxigenic endocarditis clone ST885. The *spa*-type pili are adhesins that play a major role in host cell invasion (*50*). Genomic data can identify toxin variants and predict the extent to which amino acid mutations might affect virulence and vaccine toxoid match (*36*). None of the toxin variants in our isolates harbored mutations likely to cause vaccine escape.

Our findings help elucidate *C. diphtheriae* disease epidemiology, pathogen characteristics, and transmission networks in South Africa. The high case-fatality ratio and ongoing circulation of toxigenic strains among asymptomatic carriers and cutaneous diphtheria patients stresses the importance of notifying all suspected and laboratory-confirmed cases and implementing prompt public health action and treatment to reduce transmission and death. Improved DTP vaccination coverage and improved coverage for booster doses is urgently needed and aligns with the life-course immunization model, which promotes the idea that prevention is better than cure by vaccinating persons throughout their lifespan.

Appendix 1 Metadata for study of *Corynebacterium diphtheriae* infections, South Africa, 2015–2023.

Appendix 2 Additional information for *Corynebacterium diphtheriae* infections, South Africa, 2015–2023.
