# Supplementary material for: Corynebacterium diphtheriae Infections, South Africa, 2015–2023
Source: Emerg Infect Dis. 2025 Mar;31(3):417–26. doi: 10.3201/eid3103.241211 (PMC11878320; doi:10.3201/eid3103.241211)
Supplement: Appendix 2 — Additional information for Corynebacterium diphtheriae infections, South Africa, 2015–2023. [file 24-1211-Techapp-s2.pdf]

Article DOI: https://doi.org/10.3201/eid3103.241211

EID cannot ensure accessibility for supplementary materials supplied by authors. Readers who have difficulty accessing supplementary content should contact the authors for assistance.

Corynebacterium diphtheriae Infections, South Africa, 2015–2023

Appendix 2

Appendix 2 Table 1. Pairwise SNP distances among *Corynebacterium diphtheriae* outbreak-associated lineages, South Africa, 2015–2023*

Sequence type	No. isolates	Infection category	Year	Province	No. SNP differences
ST378	29	Respiratory diphtheria, n = 20; cutaneous diphtheria, n = 2; asymptomatic carriage, n - 7	2015–2018, 2020	Kwazulu-Natal, n = 24; Western Cape, n = 4; Eastern Cape, n = 1	4–99
ST906	12	Respiratory diphtheria, n = 5; asymptomatic carriage, n = 7	2023	Western Cape	5–22
ST885	11	Endocarditis, n = 8; cutaneous diphtheria, n = 2; asymptomatic carriage, n = 1	2017, 2021–2023	Western Cape	7–36†

*SNP, single-nucleotide polymorphism; ST, sequence type.
†One isolate from an endocarditis patient reported in 2017 differed by 1,632–1,646 SNPs compared with the other ST885 isolates.

Appendix 2 Table 2. Presence of *spuA* and virulence-associated genes among toxin-positive and toxin-negative *Corynebacterium diphtheriae* isolates, South Africa, 2015–2023*

Functional gene system	Functional category	Toxin positive, n = 42	Toxin negative, n = 42	
		spuA+, n = 0	*spuA*+ , n = 20/42 (48)	*spuA*–, n = 22/42 (52)
spaA-type pili	adhesin	0	20/20 (100)	17/22 (77)
spaH-type pili	adhesin	0	15/20 (75)	5/22 (23)
spaD-type pili	adhesin	0	18/20 (90)	15/22 (68)
chtAB	heme iron uptake	0	20/20 (100)	17/22 (77)
frgCBAD	iron uptake	42/42 (100)	20/20 (100)	22/22 (100)
ciuABCD	iron uptake	42/42 (100)	20/20 (100)	22/22 (100)
ciuEFG	siderophore biosynthesis	42/42 (100)	20/20 (100)	22/22 (100)
irp6ABC	iron uptake	42/42 (100)	20/20 (100)	22/22 (100)
irp2ABCDEFGHI	siderophore biosynthesis	42/42 (100)	5/20 (12)	5/22 (23)
irp2JKLMN	iron uptake	42/42 (100)	5/20 (12)	5/22 (23)
htA-hmuTUV-htaBC	heme iron uptake	42/42 (100)	20/20 (100)	22/22 (100)
iusABCDE	iron uptake	3/42 (7)	1/20 (5)	2/22 (9)
sapADE	adhesin	42/42 (100)	20/20 (100)	22/22 (100)

*Values are no./total no. isolates (%) for each column. Total number of isolates analyzed was 84. Presence of *spuA* gene is a proxy for assigning the biotype biovar gravis; absence of *spuA* is associated with biovar mitis. +, positive; –, negative.

Appendix 2 Table 3. Presence of virulence-associated genes among outbreak-associated *Corynebacterium diphtheriae* clones ST378, ST906, and ST885, South Africa, 2015–2023*

Functional gene system	Functional category	ST378, toxin positive, respiratory diphtheria, n = 29	ST906, toxin positive, respiratory diphtheria, n = 12	ST885, toxin negative, endocarditis, n = 11
spaA-type pili	adhesin	0	0	11 (100)
spaH-type pili	adhesin	0	0	0
spaD-type pili	adhesin	0	0	11 (100)
chtAB	heme iron uptake	0	0	11 (100)
frgCBAD	iron uptake	29 (100)	12 (100)	11 (100)
ciuABCD	iron uptake	29 (100)	12 (100)	11 (100)
ciuEFG	siderophore biosynthesis	29 (100)	12 (100)	11 (100)
irp6ABC	iron uptake	29 (100)	12 (100)	11 (100)
irp2ABCDEFGHI	siderophore biosynthesis	29 (100)	12 (100)	0
irp2JKLMN	iron uptake	29 (100)	12 (100)	0
htA-hmuTUV-htaBC	heme iron uptake	29 (100)	12 (100)	0
iusABCDE	iron uptake	29 (100)	12 (100)	11 (100)
sapADE	adhesin	29 (100)	12 (100)	11 (100)

*Values are no.(%) for each sequence type. Total number of clones was 52. ST, sequence type.
